# From Molecular Flavor Signatures to Mechanism-Oriented Food Quality: Advances in Flavoromics, Fermentation Ecology, Functionality, and Safety

**DOI:** 10.3390/foods15132337

**Published:** 2026-07-01

**Authors:** Dongrui Zhao, Jianan Zhang

**Affiliations:** 1 Key Laboratory of Geriatric Nutrition and Health, Ministry of Education, Beijing Technology and Business University, Beijing 100048, China; 2 Key Laboratory of Brewing Molecular Engineering of China Light Industry, Beijing Technology and Business University, Beijing 100048, China; 3 National Baijiu and Huangjiu Research Institute, Beijing Technology and Business University, Beijing 100048, China; 4 China Food Flavor and Nutrition Health Innovation Center, Beijing Technology and Business University, Beijing 100048, China; 5 National Engineering Research Center for Rice and By-Product Deep Processing, School of Food Science and Engineering, Central South University of Forestry and Technology, Changsha 410004, China; fez.jianan@foxmail.com

Food quality is now understood less as a single attribute than as an integrated system of flavor perception, nutritional value, microbial ecology, processing history, and safety assurance. This shift is especially visible in fermented foods, brewed beverages, fresh fruits and vegetables, botanical seasonings, and rapidly perishable animal products, where volatile and non-volatile metabolites interact with microbial succession, matrix composition, and consumer perception. The papers collected in this Special Issue reflect this transformation. Together, they move from descriptive quality evaluation toward mechanism-oriented food science, in which instrumental analysis, sensory science, multi-omics, chemometrics, and process control are combined to explain why foods taste, smell, age, ferment, and deteriorate as they do. Recent advances in sensomics and flavoromics emphasize that meaningful flavor research should connect molecular identification with sensory prediction, while machine-learning-assisted flavoromics is becoming an important strategy for defining characteristic food flavor signatures [1,2].

Fermented and brewed matrices form one of the major threads of this collection. The study on lager beers demonstrates the value of combining GC-MS, GCxGC-TOF-MS, GC-O-MS, sensory evaluation, odor and taste activity analysis, multivariate statistics, and flavor addition experiments to distinguish brand-specific aroma profiles at the molecular level. Rather than treating beer flavor as a simple sum of abundant volatiles, this work highlights the importance of sensory-active trace compounds and their combined effects. A parallel focus appears in the contributions on Baijiu. The study of sauce-aroma high-temperature Daqu from the Chishui River Basin links trace volatile fingerprints, sensory scoring, molecular docking, and bacterial and fungal community structures, thereby providing a more integrated explanation of Daqu quality. The work on high- and low-alcohol Jiangxiangxing Baijiu further shifts attention from orthonasal aroma alone to retronasal perception, showing that differences in olfactory thresholds and dose-over-threshold distributions can explain why low-alcohol products may lose aroma richness even when compound concentrations appear similar. These studies echo the broader consensus that the Baijiu ecosystem is driven by complex microbial communities, enzymatic functions, and metabolite interactions [3,4,5].

Microbial succession and starter ecology are another central theme. The research on sour bamboo shoot fermentation clarifies how organic acids, especially lactic and acetic acids, help drive the transition from Enterobacteriaceae-dominated early communities to lactic-acid-bacteria-dominated fermentation systems. This finding is important not only for traditional vegetable fermentation, but also for the broader question of how microbial stress tolerance, interspecies interactions, and acidification jointly shape safety and flavor. The review on Chinese traditional fermentation starters (Qu) provides a wider technological context by organizing preparation parameters, microbial diversity, and food applications across Daqu, Xiaoqu, Hongqu, wheat bran or Jiangqu, Douchi Qu, and Furu-related starters. The companion review on ecological risks during Daqu storage extends this perspective from starter preparation to post-production stability, emphasizing that environmental disturbances can alter microecological balance, enzyme activity, and aroma formation. These contributions align with recent multi-omics research showing that precise characterization of microorganisms and their functional pathways is essential for understanding fermented food quality and for designing controlled microbial processes [6,7].

The Special Issue also broadens flavor science beyond fermented systems into fresh and processed plant-derived foods. Studies on cucumber, melon, Younai plum, Citri Reticulatae Pericarpium, and fried Tengjiao oil demonstrate how cultivar, origin, drying or storage conditions, and raw-material state can reshape volatile profiles, nutritional traits, and sensory attributes. In cucumbers, HS-SPME-GC-MS and chemometrics reveal cultivar-dependent volatile signatures and nutritional variation. In melons, multi-omics integration connects saline-alkali cultivation with changes in volatile and non-volatile metabolites, especially pathways related to flavonoid biosynthesis. The Younai plum study builds a baseline for linking sugar-acid composition, aroma-active compounds, and untargeted metabolomics. The work on Citri Reticulatae Pericarpium combines GC-MS, GC-IMS, electronic nose, sensory evaluation, and microstructure analysis to show how controlled drying and storage support volatile retention and aroma complexity. Meanwhile, the Tengjiao oil study uses molecular sensory science, AEDA, OAV analysis, recombination and omission tests, and chiral analysis to decode the flavor differences between oils prepared from fresh and dried raw materials. These examples show why modern food flavor analysis increasingly depends on complementary techniques such as GC-O-MS and GC-IMS, which can connect chemical fingerprints with sensory relevance more effectively than single-platform profiling [8,9].

Functionality and safety provide the final axis of the collection. The study on strong-flavor-type Baijiu uses widely targeted metabolomics and network pharmacology to screen potential bioactive compounds and predicted biological targets, thereby expanding Baijiu research from volatile aroma chemistry into possible functional components. Although such computational predictions require further biological validation, they provide useful hypotheses for future work on the relationship between complex food matrices and health-relevant small molecules. The contribution on cold-chain fish samples addresses a different but equally important problem: rapid safety screening. By developing a portable ion trap mass spectrometry method with nano-electrospray ionization for biogenic amines, the study responds to a long-standing need for fast, field-adaptable quality control in perishable aquatic products. This is particularly relevant because histamine and other biogenic amines are widely recognized as important food safety and freshness indicators, and their determination has remained an active analytical challenge [10].

Taken together, the articles in this Special Issue suggest several priorities for the next stage of food flavor, fermentation, and safety research. First, sensory validation should remain central. Compound annotation, even at high resolution, becomes most useful when linked to orthonasal and retronasal perception, temporal sensory dynamics, thresholds, recombination, and omission experiments. Second, multi-omics datasets should be connected more directly to process variables, microbial functions, and industrial decision points. This will allow researchers to move from correlation toward controllable mechanisms. Third, standardized data structures, shared reference materials, and transparent chemometric or machine-learning workflows will be needed if flavoromics is to support reliable prediction across varieties, origins, production batches, and storage conditions. Finally, practical translation should be kept in view. The ultimate value of these approaches lies in helping producers design foods that are flavorful, safe, nutritionally meaningful, culturally distinctive, and technologically reproducible. By bringing together work on brewed beverages, traditional starters, fermented vegetables, fruits, botanical seasonings, functional components, and rapid safety detection, this Special Issue provides a timely snapshot of a field that is becoming more integrated, more quantitative, and more capable of linking food chemistry with real eating experience.

**Frontier Perspectives**

Looking forward, food flavor, fermentation, functionality, and safety research is expected to move further toward predictive, causal, and application-oriented science. One important frontier is the construction of sensory-guided flavoromics systems that can link molecular fingerprints with real human perception. Future studies should not only identify volatile and non-volatile compounds, but also clarify how these compounds contribute to orthonasal aroma, retronasal aroma, taste, mouthfeel, aftertaste, and temporal sensory changes. Threshold determination, recombination and omission experiments, time-intensity analysis, and consumer-oriented sensory validation will remain essential for distinguishing chemically detectable compounds from truly sensory-relevant quality markers.

A second frontier lies in the deeper integration of multi-omics data with microbial ecology and process engineering. In fermented foods and brewed beverages, microbial communities do not simply coexist with flavor compounds; they actively drive substrate transformation, enzyme production, metabolite accumulation, and quality differentiation. Therefore, future research should connect metagenomics, metatranscriptomics, metabolomics, proteomics, and sensory data with fermentation parameters such as temperature, oxygen exposure, raw-material composition, starter preparation, storage environment, and process duration. Such integration will help researchers move beyond correlation-based interpretation and toward controllable mechanisms that can guide industrial production.

Artificial intelligence and machine learning will also become increasingly important in food quality prediction. Data-driven models can help screen key flavor markers, classify origins and varieties, predict sensory attributes, optimize fermentation conditions, and detect quality deterioration at an early stage. However, the reliability of these models will depend on standardized datasets, transparent feature selection, external validation, and interpretable algorithms. Future work should avoid treating machine learning as a purely statistical tool and instead use it as a bridge between chemical composition, biological function, sensory perception, and process control.

Another promising direction is the development of portable, rapid, and on-site analytical technologies. For highly perishable foods such as aquatic products, meat, fresh produce, and fermented products under storage, laboratory-based analysis alone may not meet the needs of real-time quality management. Portable mass spectrometry, electronic nose systems, electronic tongue systems, biosensors, spectral imaging, and intelligent detection platforms may provide faster tools for monitoring freshness, safety risks, microbial spoilage, and flavor deterioration. When combined with chemometrics and cloud-based databases, these technologies could support more efficient quality control across production, storage, transportation, and consumption.

Finally, future food quality research should pay more attention to translation from scientific discovery to practical food design. The ultimate goal is not only to explain why foods differ in flavor, safety, or functionality, but also to help producers design products that are stable, safe, flavorful, nutritionally meaningful, culturally recognizable, and acceptable to consumers. This requires closer collaboration among food chemists, microbiologists, sensory scientists, data scientists, process engineers, nutrition researchers, and industry practitioners. Through this interdisciplinary framework, food quality science can evolve from post hoc evaluation toward intelligent prediction, precision regulation, and consumer-centered innovation.

## Data Availability

Not applicable.
